# Controversial Topics in Animal Welfare in Latin America: A Focus on the Legislation Surrounding the Human-Companion Animal Relationship and Animals Used for Recreational Practices

**DOI:** 10.3390/ani13091463

**Published:** 2023-04-25

**Authors:** Daniel Mota-Rojas, Ana Strappini, Alexandra L. Whittaker, Marcelo Ghezzi, Cristiane Gonçalves Titto, Néstor Calderón-Maldonado, Patricia Mora-Medina, Adriana Domínguez-Oliva, Jocelyn Gómez-Prado, Ismael Hernández-Ávalos, Nancy José-Pérez, Alejandro Casas-Alvarado, Agustín Orihuela

**Affiliations:** 1 Neurophysiology, Behaviour and Animal Welfare Assessment, DPAA, Xochimilco Campus, Universidad Autónoma Metropolitana, Ciudad de México 04960, Mexico; mvz.freena@gmail.com (A.D.-O.); jocelyn.gomez.ilp@gmail.com (J.G.-P.); nancyjosenutricion2017@gmail.com (N.J.-P.); ale0164g@hotmail.com (A.C.-A.); 2 Wageningen Livestock Research, Wageningen University & Research, 6708 WD Wageningen, The Netherlands; ana.strappini@wur.nl; 3 School of Animal and Veterinary Sciences, Roseworthy Campus, University of Adelaide, Roseworthy, SA 5116, Australia; alexandra.whittaker@adelaide.edu.au; 4 Animal Welfare Area, Faculty of Veterinary Sciences (FCV), Universidad Nacional del Centro de la Provincia de Buenos Aires (UNCPBA), University Campus, Tandil 7000, Argentina; ghezzi@vet.unicen.edu.ar; 5 Laboratório de Biometeorologia e Etologia, FZEA-USP, Faculdade de Zootecnia e Engenharia de Alimentos, Universidade de São Paulo, Pirassununga 13635-900, Brazil; crisgtitto@usp.br; 6 Ethology, Bioethics and Animal Welfare, Universidad La Salle, Bogotá 110231, Colombia; nestor.calderon@gmail.com; 7 Facultad de Estudios Superiores Cuautitlán, Universidad Nacional Autónoma de México (UNAM), Cuautitlán 54714, Mexico; mormed2001@yahoo.com.mx (P.M.-M.); mvziha@hotmail.com (I.H.-Á.); 8 Facultad de Ciencias Agropecuarias, Universidad Autónoma del Estado de Morelos, Cuernavaca 62209, Mexico; aorihuela@uaem.mx

**Keywords:** welfare, Mexico, Colombia, Chile, Argentina, Brazil, companion animals

## Abstract

**Simple Summary:**

In several countries, there are practices, ideologies, rooted traditions, or legal gaps that represent challenges to animal welfare. These particularly relate to the consideration of the welfare domains of nutrition, environment, health, behavior, and corresponding mental state. In this article, we discuss some of the challenges with respect to animal welfare that arise in Latin America. Controversial topics concerning aesthetic and management practices in small animals and with the keeping of wildlife in Latin American regions are discussed. The focus is on the legal and ethical aspects, as well as the current efforts, that these countries are making to address and incorporate global welfare standards into domestic and wild animal practice and regulation.

**Abstract:**

Animal welfare is a societally relevant issue that is globally attracting increased attention. This is in addition to the importance placed on welfare for the animals themselves. However, the content and application of laws protecting animals’ welfare vary across countries. In Latin America, there are a range of common practices or activities involving certain animal species, many of which are legal, that can impair an animal’s quality of life. These include the performance of aesthetic surgical procedures; bull-, cock-, and dog fighting; and the existence of circuses that exhibit animals. The extent and impact of these practices being dependent on the socioeconomic, cultural, territorial, and regulatory landscape of each country. Particularly, Ibero-American regions face welfare challenges that might be influenced by traditions and relevant legal gaps. The objective of this article is to review controversial practices carried out in companion and entertainment animals in Latin America, with a focus on legal aspects, as well as the current efforts being made to address and incorporate global welfare standards into domestic and wild animal practice and regulation.

## 1. Introduction

Animal welfare (AW) is a relatively novel concept finding its roots as a science within the last century, with antecedents in older times when ancient philosophers pondered the emotions, rationality, and cognition of nonhuman animals [1]. It is generally considered that AW constitutes a fundamental element of modern civilizations due to the responsibility that humans often assume for animals. The most widely accepted definition of welfare was stated in 1986 by Professor Donald Broom [2], who defined “the state of an animal with respect to its attempts to confront its environment”. In 2018, the WOAH (formerly OIE) proposed a definition that elaborates on Broom’s concept. The WOAH’s proposal holds that “animal welfare designates the physical and mental state of an animal in relation to the conditions in which it lives and dies” [3]. This definition implies that an AW state comprises the integration of physical, behavioral, and mental–emotional aspects.

In an attempt to enforce and protect animal welfare, countries in Latin America and across the world have included provisions around welfare within law. Ideally, legislation on AW would reflect science and ethical viewpoints and approach issues of welfare in a multidisciplinary manner [4]; however, legislators must also consider regional influences and traditions. The diversity among Latin American countries is great in terms of geography, climate, traditions, and sociocultural conditions and, therefore, it is necessary to address the AW regulatory framework within the context of each country. As an example of this diversity, in some countries in the region cockfighting is still a common practice, in others the inclusion of animals as sentient beings in the country’s constitution is being discussed [5,6]. It is also important to consider that the written law is just one aspect of animal protection; laws are only as good as their ability to be enforced, so while some jurisdictions’ laws may seem at face value to be superior based on content, if they are not enforced then these countries may not actually be performing any better than those countries with less welfare-friendly laws.

In Latin American, there has been an escalation in the dog and cat population (an increase of 17% by 2022) [7]. This finding suggests the worth of companion animals to society and should influence legal reform and resourcing of enforcement around certain practices. However, this has not occurred universally. For example, although ear cropping and tail docking are considered unnecessary mutilations and are banned in countries such as Brazil [8], in other countries they are still practiced due to the lack of law enforcement [9,10].

Likewise, strong cultural- and tradition-related activities, such as animal fighting—including dog-, cock-, and bullfighting—are arguable events that are defended by some sectors of society (e.g., entrepreneurs and organizers) [11] and are still practiced in several countries in Latin America [12]. On the contrary, a vast majority of animal rights advocates consider these blood sports as an act of animal abuse and defend laws and regulations towards a ban on animal fighting [13].

This article reviews the ethical and legal aspects of companion and recreational animal practices in Latin American countries. The focus is on companion animals due to the fact of current societal concern and their close bond with humans [14]. The objective of this article was to review the current issues and controversial common practices that can compromise the welfare of companion animals and animals used for entertainment in Latin America.

## 2. The One Health/One Welfare Approach and Its Challenges in Latin America

The perspective of AW as an integral aspect has led to the emergence of the concept of “One Health”, an initiative developed by the World Organization for Animal Health (OIE), which recognizes that “*the health of animals and people depends on the environmental health of the ecosystem where they exist*” [15]. Later, the “One Health Initiative” adopted by the American Medical Association (AMA) and American Medical Veterinarian Association (AVMA) transformed into the “One Welfare” concept [16]. Both strategies call for implementing programs, policies, legislation, and scientific research in all sectors related to public health, animal health, and environmental care [17].

The One Health/One Welfare strategy seeks, first, to understand the fundamental nature of welfare, including cognition, emotion, environmental and organic physiology, pathologies (including zoonosis), and nutrition. Second, it utilizes AW indicators, such as physiological biomarkers (e.g., cardiorespiratory and metabolic functions), immunological and endocrine signals (e.g., stress hormones), and behavioral patterns to establish the relationship between an animal’s homeostasis and its environment [1]. Third, in conjunction with this strategy, it analyzes practice-oriented character traits—that is, handling methods—to determine whether they comply with standards that optimize AW and health, the sustainability of the environment, and public health [18].

In order to evaluate AW in a multidimensional manner, three fundamental approaches are generally used: a focus on correct organic and physiological functioning; a focus on naturality that assesses whether animals have the conditions required to express behaviors typical of their species; and the emotional focus, that ponders the sentience of nonhuman animals and how people perceive them depending on the species [19,20].

The interest in human–animal interactions and their impact on the environment and nonhuman animals has led to the One Health/One Welfare strategy, since human beings’ intentional or nonintentional actions can harm flora and fauna. In companion animals, the One Welfare approach has helped identify potential health hazards posed by dogs or cats in relation to rabies [21]. Rabies was prevalent in Brazil, Colombia, Peru, Venezuela, Nicaragua, Dominican Republic, and Mexico between 1995 and 2005, and it remains an issue today. A projection made by Yoder et al. [22] acknowledges that to eliminate human death by rabies by 2030, there needs to be the successful implementation of vaccination campaigns for companion animals in these countries. Although cases of canine and human rabies have decreased by 90% in Latin America, poverty and other public health issues strongly associate with rabies prevalence (e.g., Bolivia or Guatemala) and need addressing alongside any vaccination strategy [23].

Within Mexico, Argentina, and Chile, there has been a growing interest in teaching the concept of One Health not only in veterinary schools [24] as part of a postgraduate course for animal health professionals [25] but to society as well. Most of this is conducted through promotional videos highlighting the importance of One Health as a sustainable strategy [26] and through the delivery of e-learning courses in different languages. These have been particularly relevant with the recent pandemic, where the principles taught assist people in understanding the role of this approach to prevent future pandemic situations where human, animal, and environmental health may be compromised [27]. In Mexico, One Health is considered a part of the 2030 schedule inside the objectives of sustainable development worldwide [28]. In Argentina, the role of veterinary services in facing issues related to One Health, such as food security and safety, includes programs for the control of zoonotic pathogens that could be present in companion animals [29]. In other regions, such as Chile, the One Health Day symposium has included climate change and its effect on biodiversity, disease transmission, and bacterial resistance as a top priority [30]. Similarly, Peru uses this approach to propose vigilance and preventive measures against rabies by establishing multidisciplinary actions aimed to research the interactions between public health and animal/environment issues and implement new technologies to improve these programs [31]. This is an example of how One Health has also helped to expand science and research in these countries.

The “science of animal welfare” emerged in this context as a field that evaluates by means of a broad range of tools (biomarkers, animal-based indicators, etc.) the quality of animals’ lives (QOL) [18,32,33]. On the other hand, animal welfare legislation underpins human relationships with animals through regulating conduct towards them. It is generally considered that animal welfare law should align with the latest scientific evidence, as well as current societal viewpoints, relating to the sentience and treatment of animals. Recent legal reforms around controversial practices within Latin America serve as examples. First, there has been a recent ban on tail docking and ear cropping in Mexico in 2020 by modifying the Federal Law on Animal Health and the General Law on Ecological Balance and Environmental Protection [34]. The second example is the Chilean Law Project that aims to modify the Law of Responsible Pet Ownership and Companion Animals in 2021 to state tail docking and ear cropping as banned instead of just categorizing them as “events that can cause pain and affect animal health” according to veterinarians and the available information on pain [35].

The following sections discuss the practices that may cause potential unnecessary suffering to animals with reference to the connection (or lack of) between current animal welfare science understandings around them and the legal position on performing them in Latin America. The issues presented are those that relate to companion animals and animals used for entertainment.

## 3. Companion Animals

### 3.1. Tail Docking (Caudectomy)

Tail docking is the amputation of part or all of an animal’s tail [36]. The practice has been justified in certain circumstances due to the fact of several purported (actual or perceived) benefits. Some consider tail docking to prevent rabies, reducing the possibility of dogs being bitten in said area [37] or the acquisition of lesions during hunting [38], while others believe that it increases an animal’s speed. In earlier times, people cut off their dogs’ tails to simulate working animals and avoid paying taxes that were imposed on pets [39,40]. Today, one-third of purebred dogs are caudectomized due to the fact of this tradition [41], to prevent injury [38,42], ease mating or improve hygiene in the genital area [43], for simple convenience [44], for aesthetic purposes to enhance the animal’s bearing, or to increase aggression; since the tail conveys crucial information, docking decreases the effective interaction of dogs with each other [38].

Legislation around tail docking and conditions on its use vary across Latin America. In some states, this practice is banned. This is the case in Tamaulipas in Mexico, where the Law of Animal Protection was modified to ban docking. Modifications have also been carried out in the Federal Law on Animal Health and the General Law on Ecological Balance and Environmental Protection to consider caudectomy and otectomy (removal of all or part of the ears) as offenses within the Criminal Code [34]. Nonetheless, while this practice may be banned in written law, these provisions are not enforced uniformly throughout the territory, and may be being performed by personnel who do not have a veterinary license. Moreover, in spite of the regulation, veterinarians continue to perform the procedure when it is necessary to preserve a dog’s life. In Chile, a study by Morales et al. [45] determined that in the Province of Santiago, out of 202 surgeries, tail docking represented approximately 4% of the surgeries performed on females and 9.4% on males. In the mentioned study, it was not specified whether the procedures were performed on purebred dogs. Caudectomy is particularly seen as a practice to preserve the characteristics of pure breeds [36]. Whatever the reason, the cutting of any dermal, nervous, or vascular structure entails pain that, in most cases, is unnecessary and in no way benefits the animal.

Proponents of the procedure posit that performing this surgery in 2–5 day old puppies (usually without any anesthesia or analgesia) avoids pain. This is based on observations of the puppies continuing to suckle (considered a behavior that indicates good “welfare”) [46]. However, studies have demonstrated that pain pathways in puppies are mature at birth [38], that newborns are anatomically and physiologically structured to perceive pain [36], and that behaviors such as suckling can release endogenous opioid peptides that, in the case of puppies subjected to tail docking, could act as a mechanism of nociceptive modulation when tissue damage occurs [44,47,48]. Despite the ongoing debate as to whether caudectomies actually reduce the risk of suffering lesions, some countries maintain practices of this kind for nontherapeutic purposes [43], not only on companion animals but also on farm animals [49,50,51,52].

Another example is Ecuador, although aesthetic mutilation (i.e., nontherapeutic) in companion animals is banned (Article 8 of Chapter II of the Ordinance of Possession, Protection, and Control of Urban Fauna) and cannot be performed by veterinarians or other personnel, its practice continues to be carried out. Even though caudectomy is prohibited in some parts of Latin American countries, such as Mexico, it still occurs. The reason may be due to the insufficient supervision and ineffective law enforcement or because its regulation is limited to a specific state or region within a country, permitting, in the case of Ecuador, to be freely practiced outside the capital Quito [9].

### 3.2. Ear Cropping (Otectomy)

The partial or total extirpation of the ears (otectomy) has generated broad controversy, since the procedure has become to be considered as a form of mutilation. The World Small Animal Veterinary Association (WSAVA) classifies these surgeries as acts of mutilation because they affect animals’ sensitive tissues, bone structures, and communication appendages.

Ear cropping involves removing the auricular pavilion and then applying splints to keep the animal’s ears in an erect position. It requires a general anesthetic, as well as good surgical skills, and is therefore an act of veterinary surgery. In dogs, it is performed much more often for aesthetic than therapeutic reasons, although proponents often justify it for breeds of fighting dogs and shepherd breeds based on the argument that it reduces the incidence of bites and wounds to the ear region. Others believe that ear cropping prevents otitis and otohematomas in dogs with droopy ears [40], but there is no scientific evidence to suggest that it is an effective preventive technique [38]. There is, however, good evidence of its negative impacts, such as allowing dust, dirt, and other foreign matter to reach the eardrum [53], as well as, obviously, causing postsurgical pain because the ears are densely innervated areas that bleed easily [41].

These surgical procedures performed on dogs for “aesthetic” purposes [38,54], cause controversy in Latin American society [55]. In Brazil, aesthetic ear cropping is considered a crime and a form of animal abuse. However, it is acceptable to perform cuts in the ears of neutered free-roaming cats to signal that the animal is already neutered [8]. In Colombia, this practice is objected to by veterinarians and animal rights activists. This situation has also led to professional usurpation that is harmful to the health of animals when surgical procedures end up being performed by nonauthorized personnel [10].

The Chilean Veterinary Medical Association (Colmevet), through its National Technical Commissions on Responsible Pet Ownership, Bioethics and Animal Welfare, expressed its absolute rejection of cosmetic surgeries performed on animals and urged that these aesthetic surgeries be banned in Chile. In relation to it, in 2019, the Chamber of Deputies proposed a draft law (1394-D-2019) that banned mutilations on nonhuman animals, except where they were performed by veterinary professionals for justifiable causes. This notwithstanding that action is being taken at a local level. For example, since July 2020 the Argentinian Kennel Club has ruled that dogs with cropped ears or docked tails are not eligible for any kind of champion title in any discipline and will not receive breeding qualification unless a veterinary certificate justifying performance of the procedure is presented [56]. In other countries, such as Uruguay, changes have been made more gradually. The Uruguayan Kennel Club has ruled that “from 1st January 2025, dogs with amputated ears/tail, belonging to breeds for which the standard does not refer to amputated ears/tail, may no longer be entered in any beauty show where the standards of the federation are used”.

Although the enforcement of the law varies from country to country, and even within the same country, a common factor in Latin America is the growing public concern around AW issues that is pushing legislators’ decisions to ban or regulate these practices.

### 3.3. Removal of Vocal Cords in Dogs (Cordectomy)

Dogs communicate in diverse ways, including through the use of body language, facial expressions, and vocalizations [57]. In general and with the exception of the Basenji breed (whose vocal cord structure is narrower and flatter than other breeds) [58], all dogs use vocalization to communicate intra- and interspecifically [59,60]. Despite this important social function, some owners have their dogs’ vocal cords removed partially or totally to soften or eliminate their barking, even though there is no scientific justification for the practice. Excessive vocalizations, or hypervocalization, in canines can be a manifestation of several pathologies such as anxiety, frustration, and pain. Therefore, devocalization in these cases does not treat the underlying etiology of the condition, seriously impacting the welfare of the animal [41,57].

In Mexico, the Senate of the Republic, in 2015, stated that surgeries such as cordectomy in dogs had increased due the increased public health problem of excessive noise in the urban sectors of the country [61]. The Senate proposed a reformation of the Federal Animal Health Law to include the term “animal welfare” and to consider the cutting of vocal cords and declawing in cats as crimes and animal abuse [62]. This is in spite of the practices of cordectomy, caudectomy, and otectomy being banned in other states of the country, such as Coahuila, since November 2013 [63]. This illustrates the fragmented nature of animal welfare law in this jurisdiction. A similar situation arises in the Argentinian province of Buenos Aires, where cordectomy is banned among other mutilations in domestic animals [64], but this remains to be the case in other provinces.

Unfortunately, the lack of organized data collection on the incidence of procedure performance renders it difficult to determine the extent of the issue or the impact of any legal reform [65].

### 3.4. Declawing of Cats (Onychectomy)

The onychectomy procedure consists of the surgical amputation of the third phalanx in both fore- and hindlimbs to prevent the claws from growing back [66,67]. The supposed benefit of carrying out this procedure is to avoid behavioral problems that are not desired by the owner, such as the destruction of furniture [68,69]. Scratching is a natural behavior in cats, useful for visual and chemical communication. After scratching, cats usually mark their territory through chemical signals released by the footpads and through visual markings on the scratched surface [70]. Since this behavior cannot be eliminated, treatment focuses on redirecting scratching to surfaces designed for it, such as scratching posts. Although nontherapeutic onychectomy was suggested to avoid euthanasia or abandonment, current research has shown that scratching behavior is an uncommon reason for shelter relinquishment and a ban on elective onychectomy did not increase shelter relinquishment or euthanasia [71].

Declawing can cause severe acute pain due to the fact of injury to the tendons, dermis, and muscles, which has repercussions on cats’ well-being and health status [72]. In this sense, Curcio et al. [73] evaluated the effectiveness of the use of buprenorphine alone or in combination with bupivacaine in 20 cats undergoing onychectomy. The authors found that, despite using these treatments, six animals required analgesic rescue, showing the intensity of the pain caused by this handling.

Furthermore, its practice is debated because of its possible adverse effects. Short-term effects include pain perception in 50% of patients, lameness, bleeding, behavioral changes, lethargy, decreased appetite, tissue necrosis, and cystitis [67]. Long-term effects include claw growth, chronic pain, leg pain, flexor tendon contraction, and increased biting [74]. It is for these reasons that this practice is prohibited in Australia, New Zealand, and several European countries. Regarding Latin American countries, the Chamber of Deputies in Brazil published a project to classify this practice as animal abuse and proposed a penalty of 3 months to a year in detention plus a fine for those who carry it out [75]. Similarly, in Argentina the Senate and the Chamber of Deputies formulated project 1394-D-2019 to prohibit aesthetic mutilations in animals throughout the territory, within which declawing is contemplated [76]. In countries such as Colombia and Mexico, there are no specific proposals or laws for this procedure; however, the practice of nonmedical mutilation that seriously impairs your health or physical integrity is considered mistreatment [77,78].

### 3.5. Legislation Regarding Mutilations of Healthy Animals

In many countries, primary and secondary legislation exists that governs painful procedures. These documents typically consider that the arguments presented to defend them are insufficient to justify exposing animals to unnecessary suffering [79]. Turning to Latin American countries, Chile has an initiative to add an article to its Law for the Responsible Ownership of Pets and Companion Animals that will sanction devocalization, tail docking, and ear cropping for aesthetic purposes [80]. In Argentina, Law 14.346, Article 3, considers as “cruelty” the mutilation of any part of an animal not in response to therapeutic, health, or humanitarian conditions. In addition, surgical procedures can only be performed legally by licensed veterinary surgeons using an appropriate protocol for anesthesia [81]. In Mexico, as stated before, reforms of the Law of Animal Protection in the state of Tamaulipas now prohibit mutilations (including claw and teeth removal) for nonmedical purposes [82]. Likewise, in 2016, Colombia modified Law 84 (1989) into Law 1774, known as the National Statute for the Protection of Animals, which states that animals deserve protection from suffering and pain. Within this law, the mutilation of any living animal is classified as cruelty, with exceptions when technical, scientific, zooprophylactic, and aesthetic reasons support this decision. Additionally, Colombia considers that a route to address the social problem of animal cruelty (known as any act that intentionally causes pain and suffering to an animal) [83] is early education in adolescents promoting and prioritizing responsible pet ownership and AW [84]. This also includes veterinary professionals, who must apply animal welfare science concepts in an ethical way to promote the quality of life of companions animals, including preventing acts of cruelty [85].

In Brazil, the Law project 236/12 aims to establish sanctions beyond the administrative or educational penalties, considering them a crime. The objective is to prevent animals from undergoing ear cropping and tail docking [86]. On the other hand, in the State of Amazonas, in Brazil, Law No. 5.543 prohibited the mutilation of companion animals, imposing penalties ranging from a warning to fines up to BRL 10,000 in the case of recidivism [87]. In the case of Chile, the project to amend Law No. 21.020 proposes changes to preserve the welfare of companion animals by not performing aesthetic surgeries, banning dog fighting and animal training, and preventing abandonment [88]. This strategy of banning unnecessary surgical practices is aligned with other aspects of AW, such as abandonment, animal fighting, and unlawful spectacles that affect the behavioral repertoire of dogs and cats, which were also included in Law No. 30407 in Peru [89].

Table 1 summarizes the information regarding tail docking and ear cropping among the mentioned countries.

### 3.6. Elective Surgeries: Sterilization

Sterilization procedures for dogs and cats have increased in number since 1970. These surgeries are performed with the goal of controlling the populations of pets [110,111,112,113] and stray animals, and the public health problems they cause [114,115,116,117,118,119]. Diverse studies associate sterilization with health benefits for animals. For example, in female cats sterilized before one year of age, the risk of developing mammary neoplasia is reduced by 86% [120], while castration of males prevents testicular neoplasia [113,121]. Studies of male canines report that castration can prevent benign prostate hyperplasia, a condition observed in 95–100% of noncastrated dogs aged 9 years and over [122,123,124,125] in association with incidences of prostatitis [126,127,128,129]. Another condition in females that can be reduced by sterilization with the complete extraction of ovaries and uterus is pyometra [113], a pathology that has an incidence of 25–66% in nonsterilized bitches aged 9–10 years in such breeds as collie, golden Labrador, Labrador retriever, rottweiler, and German shepherd [130,131,132]. Likewise, it is believed that these procedures could mitigate undesirable behaviors such as aggression, especially in males, though they are not considered definitive forms of treatment [117,133].

Dawson et al. [115] describe that sterilization rates vary from 1 to 43% [134] in different countries depending on variables such as socioeconomic status [135,136], owner’s gender [137], sex of the pet [138,139], and owner’s perception of the procedure and its implications [115,140]. In countries such as the United States, Australia, and New Zealand, sterilization is now a normative or even mandated practice [140], but in several Latin American countries—México, Argentina, and Chile—it is permitted but not mandatory [141,142,143].

For example, in Yucatán, Mexico, 72.8% of people living in the cities own a dog but only 3.1% of these dogs are neutered, and dog neutering occurs at a lower rate than bitches (1.2% and 1.9%, respectively). When questioned on the reasons for not neutering, 74.6% of the owners stated that this was because of the risks and suffering that arose from the surgery [144]. Similarly, in a study performed in Brazil, sterilization was also considered an act of cruelty and a risk for death [145]. The low number of neutered animals in Mexico might also be influenced by socioeconomic factors where rural areas have less resourcing and lack public services. As a result only 1.8% of animals are spayed [112]. However, Mexico does have spay and neuter services of low or no cost in recognition that these practices contribute to the reduction of stray animals and the associated public health issues associated with these animals (e.g., rabies) [146]. Likewise, in Chile, the problem of free-roaming dogs and uncontrolled reproduction has grown from 2018 to 2020, and USD 1.5 million was spent on sterilization campaigns in remote areas or places with a high number of stray dogs [147].

As for any surgical procedure, collateral surgical damage can occur, such as urinary incontinence [148], development of lymphomas [149], and chronic pain. These effects of surgery can severely compromise the welfare of those animals. However, when surgery is performed by experienced veterinary surgeons these risks are rare (9.3–30.9% incidence of urinary incontinence if the bitch’s body weight is less than or greater than 20 kg, respectively) [38]. An alternate common reason for not neutering animals is cost related. In a study by Baquero et al. [145], 33% of respondents interviewed discussed cost as the main cause of not sterilizing pets. Additional reasons for not sterilizing pets were desire to breed, not having time to care for the animal after the surgery, or the age of the pet. In contrast, in Mexico, only a small percentage said that it was due to the cost of the surgery [144]. In Brazil, socioeconomic factors, such as income and education, have been associated to the level of responsible guardianship, where people with a higher income were also more concerned about sterilizing programs [150].

There are also differences in the sterilization rates between male and female animals, as well as in terms of species. In a survey conducted in two neighborhoods in Argentina, out of 51% of canines that had been sterilized, 31.2% were male and 72.1% female, showing a clear tendency to perform surgery on females [151]. Similarly, in a study conducted by Faver [152] using a convenience sample of 131 Latin American students who owned companion animals, a difference was observed between the acceptance to neuter/spay cats or dogs, with the procedure being more accepted in the former (60% vs. 26.4%, respectively). In this study, cat owners acknowledged the benefits of early spaying, contrary to dog owners. For the latter group, 41.4% suggested they wanted to breed from their dog, 25.7% were concerned about the cost of the procedure, and 18.6% considered spaying an unacceptable practice for companion animals. Due to the clear differences in attitudes towards both species, the authors emphasize the importance of social education on the animal and public health benefits of early sterilization [151].

In developed nations, such as Norway and Germany, as well as in Scandinavia, where the rates of stray animals are among the lowest in the world, the surgical neutering of healthy dogs is considered an unnecessary mutilation, and it is prohibited [153,154]; although other European countries that have ratified the Council of Europe Convention allow surgical procedures to prevent a pet’s reproduction [155]. Contrarily, in Latin American countries, the overpopulation of stray dogs is a serious public health problem. Mass animal sterilization programs have been proposed to manage the issue and avoid the euthanasia of approximately 4 to 10 million animals annually. Poss and Everett [156] have proposed binational projects such as the one in El Paso Country between the US and Mexico, in which, in a period of 5 months, low-cost sterilization was performed in 1108 animals (959 dogs and 149 cats). Likewise, in Mexico, nonsurgical orchiectomy with zinc gluconate has been used since 2008 as a practical and cost-effective alternative, especially in places where there are no clinical facilities to undergo a surgical procedure [157]. This technique was later regulated and used in other countries with canine overpopulation problems, such as Colombia, Panama, and Bolivia [158]. Although the goal of sterilization is to reduce overpopulation, these procedures should be accompanied by responsible ownership education based on scientific findings and adoption campaigns.

### 3.7. Mass Breeding of Companion Animals

The dog breeding market is regulated by supply and demand. It is also influenced by the popularity of certain breeds, making it a lucrative business. However, the overproduction of unwanted animals can lead to overcrowded animal shelters, euthanasia, or the killing of millions of cats and dogs each year due to the loss of interest or for economic reasons [159]. Moreover, the maintenance conditions in puppy mills are not always regulated, particularly in Latin American countries, where there is a high number of illegal breeders that prioritize profit over the health and welfare of animals, incurring animal cruelty concerns [160]. Additionally, it has been reported that pets purchased in pet stores show a higher incidence of emotional and behavioral problems (fear and aggression) compared to animals purchased from other sources [161].

Consequently, countries such as the United Kingdom, the United States, and Australia have created regulations on excessive breeding and sale by third parties, although the legal importation and kennel trading of puppies still remain unchecked [159].

In Mexico, the Animal Protection Law of Mexico City authorizes the sale of animals only in established places. This law promotes the issuance of purchase certificates and the verification and regulation of animal control centers to detect anomalies and shortcomings in legal breeders’ practices [100]. However, although companion animal breeding is regulated to some extent, in states such as Nuevo Leon the sale of pets in stores has been banned since 2023, and a similar regulation has been discussed for other states in the country to reinforce actions against animal cruelty [162,163]. However, spaces such as the Sonora market, where the unregulated sale of cats, chickens, goats, and unconventional species (e.g., monkeys, iguanas, and toucans) housed in inappropriate conditions continues. This scenario is reflected in other Latin American countries [164,165].

### 3.8. Trade of Wildlife Species Market as Exotic Companion Animals in Latin American

Within Latin America, megadiverse countries, such as Brazil, Colombia, Mexico, Costa Rica, Venezuela, Ecuador, and Peru, are characterized by having have more than 5000 species of endemic animals and plants, some of them having approximately 60 to 70% of the total number of these species around the world [166]. While this represents an advantage, the high diversity of wildlife species is one of the main reasons why these countries are involved in exotic animal illegal trade. For example, in Brazil, it is estimated that 38 million wild animals are trafficked every year [167], including fish, birds, mammals, reptiles, and amphibians [168]. In Colombia, although owning wildlife is banned, species such as the slider turtle are endangered due to the fact of illegal trade on the black market [169].

The trade of exotic animals does not only have a severe impact on wildlife conservation but poses a risk to public health and to the species that are not able to be rehabilitated and returned to the wild. Additionally, this trade is not confined to wildlife smuggling inside Latin America but extends to movements across international boundaries [170]. For example, this has been identified in Mexico, where a link between some countries of the European Union (Austria, Czech Republic, Germany, the Netherlands, Slovenia, and Sweden) and Mexican illegal trade organizations trafficked a wide range of animals, from elephants and carnivores to reptiles, among other species [171]. In this country, the inclusion of Article 419 Ter and the reformation of Article 420 of the Federal Penal Code impose sanctions (200 to 1000 days of minimum wage) and prison (six months to three years) to any person involved in wildlife trade [166]. In Brazil, efforts are aimed at classifying exotic animal trafficking as a serious crime [168].

### 3.9. Animal Cruelty and Its Relationship with Interpersonal Violence

Animal abuse and cruelty is considered both a risk factor and a potential consequence of interpersonal violence [83].

Various scientific studies have shown a connection between animal abuse and violence towards other humans [172]. For example, in Colombia and Brazil, more than 95% of small animal veterinary practitioners recognize that there is a relationship between animal abuse (which includes both physical and mental abuse) and interpersonal violence [173,174]. In fact, in recent years, veterinarians play an important role in detecting and intervening in the cycle of violence when interacting with patients and their owners in daily practice [175]. Therefore, it is essential for veterinarians to be trained to report suspected cases of animal cruelty due to the fact of their legal right to do so.

Monsalve et al. [173] showed that a significant percentage of small animal practitioners in Brazil and Colombia had suspected that their patients might have suffered animal abuse (48.1% and 64.5%, respectively), but only a small percentage of them reported it to the competent authorities (32.7% and 10.8%, respectively). In addition, it was determined that training in forensic veterinary medicine and animal welfare was deficient. This demonstrates the need to strengthen education, detection, and enforcement in cases of violence, since veterinarians have the responsibility to protect and promote the well-being of both animals and people to contribute to the “One Health, One Welfare” approach.

Organizations such as the National Link Coalition and the National Coalition against Domestic Violence assist by providing information and statistical data on pet abuse and domestic violence. This information includes relevant public policy, programs, and research aimed at addressing the topic. The United Kingdom and New Zealand have organizations such as Medics Against Violence and the Veterinary Council of New Zealand, which provide guidelines on protocols to follow in the case of suspicion or identification of animal abuse and/or domestic violence [176].

Mexico ranked third worldwide in terms of animal cruelty incidence in 2016 [177]. A complaint for physical abuse or animal abandonment can be filed with the Attorney General’s Office State Department of the Environment in the State of Nuevo León [178]. However, the enforcement of animal welfare laws can be challenging in Mexico City, which received complaints of 3663 cases of animal abuse in 2022, according to the Office of Environmental and Territorial Planning [179]. Among the organizations that support the city’s animal protection law is the animal surveillance brigade, which only has 79 members trained in handling complaints in a city that itself reports 61.4% of households with pets [180,181].

In Argentina, it is also possible to report mistreatment and acts of cruelty, which are punishable by imprisonment from 15 days to 1 year [182]. As in Brazil, complaints can be made by any citizen, preferably over 21 years of age, yet the law remains silent on the reporting of cruelty by veterinarians. On the other hand, in Colombia there is a specialized program called the “Escuadrón anticrueldad”, composed of veterinarians, technical support personnel, and lawyers who are dedicated to caring for animals as victims of cruelty [183]. Both in Colombia and Brazil, there is a law that encourages veterinarians to report cases of animal abuse [77,184]. It is important to note that one of the great challenges that Latin America is facing is socioeconomic vulnerability, where a significant percentage of animal abuse cases occur in families with economic disadvantages and are likely to be failure of the duty of care cases rather than intentional acts [185]. Additionally, mental and emotional abuse is often more difficult to identify and prosecute than physical aggression [173].

### 3.10. Quality of Life and Euthanasia in Companion Animals

Quality of life (QOL) is another concept associated with AW science. It is defined as a series of conditions that an individual animal requires to satisfy its needs, not just to ensure survival but to live with some degree of comfort [186]. In recent years, numerous definitions of quality of life in relation to animals have been developed, but it has not yet been possible to validate them due to the fact of their intimate association with equivalent terms such as happiness and joy that, up to now, have not been satisfactorily defined even for human beings [187,188].

In animals, the concept of QOL has centered on a series of internal and external factors that establish a positive balance, given that in their daily lives, animals will be exposed to negative conditions (e.g., illness, stress, and pain). Therefore, strategies to approach these issues require knowledge and the application of animal ethology and management, as well as an understanding of animals’ communication means and their perception of positive and negative emotions. Veterinarians and animal welfare scientists play a key role in evaluating QOL. Focuses for companion animals have been the development of “pet-friendly, low-stress, and fear-free” strategies and the routine use of pain assessment and management in clinics [189], particularly in chronic diseases where episodic or constant pain can significantly affect animals’ QOL and lead to altered mental states, such as fear or sensations of frustration or anxiety [187].

To aid in the evaluation of pain and conditions that significantly reduce the health of companion animals and may require a treatment or intervention, animal welfare science provides the basis for developing tools to assess QOL [188]. One of them is survey-based studies with owners (or the person who knows the companion animal best). They are based on observing the dog or cat’s behavior for a period, usually a week to a month, and evaluating criteria such as play, appetite, psychological health, and physical status, among others. Some formats allow the evaluator to assign numerical scores to these parameters to increase objectivity [190]. Surveys performed in countries such as Mexico help to analyze welfare issues such as free-roaming dogs, as well as to comprehend the main problems that arise with the overpopulation of dogs (e.g., rabies) and how society can intervene to face the problem (e.g., dog sterilization campaigns) [191]. Most, however, are designed to evaluate the consequences of a specific illness, so strict standardization or validation is not possible. Some authors recommend subjecting these instruments to validation by professional scientists and emphasize the role of veterinarians as advisers who can orient owners [192].

The controversy that surrounds QOL intensifies when it becomes necessary to determine the end point of the lives of animals with unfavorable clinical prognoses. In situations involving pets, signs of inappetence and depression have a significant impact on owners’ acceptance of the fact that the QOL of their pets is diminishing. The chronic diseases most often considered in relation to reduced QOL in animals are malignant neoplasms, cardiovascular syndromes, kidney failure, and alterations in the articulations, but another factor that influences decision making in most reported cases of declining QOL is economic [193].

Recently, a public health problem in Latin American countries, which is also seen in companion animals, is obesity. In Brazil, the prevalence of obese dogs has been estimated at 40.5%, being more frequent in females and in canines that have been neutered [194]. The problem of obesity in animals, the associated comorbidities, and the shortened lifespan can all lead to a reduced QOL. Another public health issue is attributed to the increase in populations of stray dogs in areas such as southern Mexico. These dogs represent a source of zoonotic agents, which impacts on the health of congeners, human populations, and the biodiversity of wild fauna. The presence of gastrointestinal parasites, such as *Ancylostoma caninum*, *Toxocara canis*, or *Leptospira*, produces chronic health problems associated with a reduced QOL of stray animals. Additionally, these animals are often exposed to injuries, respiratory diseases, or wounds on limbs that impede their movement and affect their welfare [195]. Due to the repercussions for health and the malnutrition of these canines, euthanasia has been suggested as a way to prevent poor AW, QOL, and foment the conservation of wild fauna [20,196]. This contrasts with countries such as Austria, Germany, Italy, and Netherlands, where euthanasia is only allowed when the animal presents with incurable diseases or severe behavioral problems that cannot be solved [155]. However, while euthanasia can reduce some of the issues associated with stray dogs, the root cause of this issue is abandonment of dogs by irresponsible owners, which will likely continue given the inefficient laws around this issue [197].

Clearly, performing evaluations of the QOL of animals demands establishing, first, a clear definition of the concept itself inside the current legislation in Latin American regions, because, although countries such as Colombia in their Law 2054 of 2020 mention elements that are implicit in QOL assessment (animal abuse, abandonment, and negligence), the concept is not mentioned per se [198]. This task requires additional studies to inform and validate the criteria used, including how objective assessments made by veterinarians can be incorporated, since these are shared decisions taken by clinical professionals and owners.

## 4. Animals Used in Entertainment

Since ancient times, animals have been used in various activities with the main objective to entertain humans. However, some sectors of society are exerting pressure to eliminate these recreational activities because of a growing perception that they compromise the welfare of animals and are, thus, unethical. In opposition, the industries often defend certain recreational practices because they consider them essential for the conservation of the species [24,199].

### 4.1. Tauromaquia (Bullfighting)

Tauromaquia is one of the most controversial practices in terms of AW [200]. Bullfighting is practiced in several countries in Ibero-America [12] in events that consist of three stages. In the first, a rider wounds an aggressive bull using a lance. In the second, the bull is challenged by a man on foot who tricks it to follow a decoy—a red cape—while spears (banderillas) are stabbed between the animal’s scapulae. In the third, the matador attempts to kill the bull by stabbing it with his sword [201]. Some sectors of society consider bullfighting part of the culture, art, and national identity of their country [202,203], but other groups see it as an especially severe form of animal abuse in which bulls are tortured until they die. Some scientific studies suggest that bulls bred for fighting (toros de lidia) secrete large amounts of endorphins during the event that help mitigate pain [204,205], but it seems obvious that the behaviors that bulls exhibit in response to those physical attacks and wounds—agitating the tail, forced respiration, exhaustion, and refusal to move [206]—indicate that they feel pain. Other studies have described the severe physical damage that bulls have after the fight, clearly revealing that this event has repercussions for the welfare of the species and constitutes a form of animal abuse [207,208,209,210]. The breeders who raise these bulls defend their trade by arguing that this species is raised exclusively to participate in this spectacle, so if bullfighting is prohibited, this breed will disappear. To date, it has not been possible to conduct scientific studies that analyze the physiological variables of bulls prior to events, because they are highly reactive animals selected, precisely, for their aggressiveness (in contrast to most domesticated species, which are selected for their docility). This constrains our ability to gather valid scientific information for decision making regarding the welfare of fighting bulls.

In several Latin American countries, bullfighting is still practiced, and it is not an activity prohibited by law. In countries such as Mexico, although people who approve of bullfighting are against animal abuse and consider other blood sports such as dog fighting as morally incorrect, tauromaquia tends to be justified even though they mutilate and kill the “toro de Lidia”. One of the reasons given by those who favor bullfighting is that the way the animal is killed is considered an “art”, and fighting bulls are bred exclusively for this purpose [211]. In this country, taking as an example the law 28/2010 of Catalonia, Spain, which prohibits bullfighting in the region, in 2011 an initiative was presented to prohibit bullfighting. In 2012, Veracruz was the first Mexican state to ban bullfighting and any other event that involved animal abuse. In other states, such as Mexico City, because Mexico’s Monumental Plaza, the country’s largest bullfighting arena, is a major source of income and employment, laws protecting domestic animals and wildlife often exclude fighting bulls [13]. A similar case occurs in Peru with Law 30407 on Animal Protection and Welfare, which is designed to safeguard animal welfare, excluding bullfights and cockfights, justifying it as a cultural exhibition [212]. This also occurs in Colombia, Bogotá [213], and in most Latin American countries, where despite the anti-bullfighting movements, the traditions and socioeconomic factors of the countries render the prohibition of bullfights a complex topic.

### 4.2. Cockfights

This event has been described as the “most ancient animal sport” [214]. This activity is carried out with fighting cocks (Aseel) are characterized by muscular legs and scaly, purple- and black-colored shins, and aggressive behavior [215,216].

In general, the countries of Latin America have changed their position on cockfighting from consideration of it as a tradition of various regions to the prohibition and penalization of it by law. In Costa Rica, in 1884 cockfighting was allowed under certain strict requirements, since it constituted a source of income for the country (in Agreement No. 89 of Costa Rica). However, by 1889, Decree No. 47 banned cockfighting, and by 1914, with the Animal Protection Society, an agreement was signed to reinforce the decision, arguing the AW of the roosters [217]. In Mexico, cockfighting is frequently associated with other illicit activities in rural areas of the country. Today, it has lost its folkloric character and has become a clandestine activity [218]. On the contrary, in Venezuela, cockfights are still carried out as recreational activities and are a source of employment for the population, especially during national holidays. Interestingly, the animals used in these activities are considered to have excellent phenotypic and genotypic traits exclusively selected to participate in fights [219]. While cocks often receive great care from their owners (similar to the care given to pets), this is given as a result of their extrinsic value being a source of revenue [220]. This care may include multiple actions to conserve their health [219,221], the biosecurity of their breeding areas [222], and maintain production processes [223]. However, once these birds reach the end of their useful life as “fighters”, there is little information available on their fate.

### 4.3. Dogfights

Dogfights are described as spectacles in which two dogs are provoked until they attack each other [24,199,224]. In spite of these events being banned by law in many countries they still often take place illegally. Two dogs (usually pit bulls) are placed in a ring and fight until one surrenders or dies. Fights end in one of four ways: one dog is declared the loser though it did not abandon the fight; an owner decides to withdraw his dog; one or both dogs die; or one jumps out of the ring [225]. Dogfights are generally regarded as cruel due to the pain and injuries inflicted. Moreover, the defeated dog has been tortured and mutilated and will likely be killed by an owner “ashamed” of its cowardice. Another aspect that has been highly criticized is the fact that owners train their dogs using strays captured in the street and/or stolen cats. Despite these elements, people in some regions of the planet defend this practice as a longstanding cultural phenomenon [226], and the sale of dog breeds considered fighting breeds is promoted or popularized, causing public health problems (e.g., attacks on children and other adults) when owners are unaware of the behavioral management required for certain breeds [227]. In this country, in 2012, the Law for the Protection of Animals of the Distrito Federal stipulated dog fights as animal cruelty, despite excluding bullfights, cockfights, and horse races. This law banned dog fights and designed penalties of one to four years in prison and fines to anyone who organizes, promotes, disseminates, or is involved in fights with or without bets [228].

In Latin America, dog fights are frequently associated with gambling and illicit activities. In Colombia, León [229] studied the socioeconomic and educational profile of dog fighters, reporting that people who participate or promote dog fighting are from different economic strata, and their education ranges from low levels to university or professional. Interestingly, these participants justify their activity by saying that dog fighting is “part of the aggressive nature and instinct of the animal”. This description has an impact on public opinion concerning the breeds used in these practices, and some people have proposed massive sterilization programs to eradicate the population of these breeds [229].

With respect to legislation governing spectacles that involve animal fights, Mexico’s Diario Oficial de la Federación [230] published a ban on dog fights in 2017. They are also prohibited in Brazil [231], Tegucigalpa, Honduras, and La Paz [232], while in Chile there are proposals to modify Article 291 BIS of the penal code to prohibit dog fights and cockfights [233]. Argentina prohibits all animal fights, public or private (Law 14.346). The province of Buenos Aires prohibits clandestine dog races but allows them in legally licensed racecourses (Law 12.499) [182,234].

### 4.4. Circuses

Circuses with animal acts appeared in Great Britain in the late 18th century, an activity that, at the beginning of the 19th century, was replicated by many other countries, including countries in America [235]. While they may employ domesticated animals, they often utilize wild species that are confined in wagons during transport and in cages before their acts [236,237]. This means that their natural behaviors are constrained, and their diets modified. In addition, they are subjected to intense training and experience periods of anxiety prior to their acts. These are some of the factors that brought circuses under close scrutiny, although few scientific studies on circus animal welfare have actually been conducted [199]. Hitchens et al. [238] analyzed information from circuses that utilized animals in the period 2010–2014. Their report concludes that 9.1% of circuses failed to comply with requirements for care on hooves, claws, and hair, and that 10% failed to satisfy the expected corporal conditions of the animals involved.

Despite the scarcity of technical–scientific information, several countries—such as the UK—have enacted laws that restrict the participation of wild animals in these events [239]. Mexico prohibits circuses from using animals [240]. This is in spite of a Mexican study [241] that found that only 56% of a group of people who usually attend circuses disagree or strongly disagree with the use of animals in the circus, while the rest either agreed or were unequivocal around their use. In Chile, a modification of existing legislation was proposed to prohibit the use of animals in circuses, based principally on the use of inhumane training methods and that animals are often forced to live with other species (prey–predator), which induces stress [242]. In Colombia, despite the fact that the presence of animals in circuses was considered part of the country’s culture [243], the Constitutional Court in Sentence C-283, in 2014, prohibited the use of wild animals [244] in fixed and traveling circuses, following Law 1638 of 2013. However, this only extends to wild animals not domesticated animals used in shows. Costa Rica, Bolivia, Nicaragua, Paraguay, and Peru have also banned the use of animals in circuses [245]. In Argentina, Law 22.421 [246] protects wild animals that participate in circuses. After its enactment in 1981, the number of circuses that used animals began to decline. By 2015, no circuses with wild animals remained.

### 4.5. Dolphinariums

As with zoos, controversy surrounds dolphinariums and aquariums [247], though the former are usually research centers that implement measures to improve their practices for the benefit of animals both captive and wild [248]. There are, however, documented cases of poor animal welfare. Indeed, it has been reported that 24% of the orcas kept in captivity show severe to extreme coronal mandibular deterioration, while over 60% have fractures in mandibular teeth 2 and 3, attributed to stereotypical oral actions such as biting the surface of the tank [249]. In contrast, Brando et al. [250] demonstrated that the welfare of bottlenose dolphins (*Tursiops truncatus*) is neither compromised nor enhanced when they participate in sessions of “swimming with dolphins”, though these were once considered stressful events for those aquatic animals.

With respect to legislation in this area, since 2005 Chile has modified its norms to prohibit the capture of cetaceans and their exhibition in captivity [251], just as Costa Rica prohibited the holding of dolphins and whales in captivity, as well as swimming with them. Mexico, which homes 8% of the world’s dolphinariums and has the largest dolphin captivity industry in all of Latin America, has prohibited the use of marine mammals in traveling shows, as well as their extraction from the wild, with the exception of those caught for research purposes by accredited institutions [252]. It should be noted that despite the heterogeneity that may exist within each country and among Latin American countries, annual congresses of the Latin American Zoo and Aquarium Association (ALPZA) have been held with the aim of ensuring that the 22 member countries of the ALPZA continue improving and ensure a good level of AW [253]. Table 2 presents a summary of advances in legislation that have occurred in various Latin American countries regarding these recreational activities involving animals and the arguments that have been expressed in favor and against them in each case.

## 5. Conclusions

In Latin America, AW regarding companion animals and animals used in entertainment presents diverse challenges and controversies.

For companion animals, surgical procedures for nontherapeutic purposes and the anthropomorphizing of dogs and cats are two elements that seriously affect the QOL of these animals. On the one hand, surgeries such as caudectomy can reduce an animal’s communication skills and compromise its social interaction with peers and even with humans, which can be dangerous. While on the other hand, tutors could be offering nonrecommended diets, which can result in health problems. Promisingly, the legislation that regulates surgical procedures in healthy animals (i.e., aesthetic surgeries) is slowly moving towards a more ethical veterinary practice. Although it can be considered that laws regarding practices (especially concerning tail docking and ear cropping) in companion animals are more advanced due to their close bond with humans, currently, topics such as anthropomorphic activities or diet selection are not mentioned in Latin American countries’ laws. In addition, the enforcement of the law in selected states of countries, such as Mexico or Colombia, impedes the compliance of the law in its entirety.

Animals used in entertainment, regardless of the species, can be subject to unnecessary painful activities. Unlike what happens with companion animals, strong cultural and/or economic aspects (both in the case of bullfighting) often hamper legal reform efforts to ban or significantly modify these events. Even animals sheltered in dolphinariums can present injuries or develop unwanted behaviors, although the difference is that the main objective of these places is not to entertain people regardless of the well-being of the animal. In terms of laws, a significant number of Latin American countries have agreed on the need to prohibit activities such as dog fighting and the use of animals in the circus; however, in practices such as bullfighting and cockfighting, opinions are divided. Countries such as Peru, Colombia, and Venezuela have given priority to the cultural aspect.

In conclusion, the achievement of optimum AW in Latin American countries requires a robust application of animal welfare science and welfare assessment as a comprehensive approach. The consideration of ethical, societal, and legal frameworks and constraints are factors that veterinarians and animal scientists require to promote AW. Teaching AW to the public and future practitioners in their discipline and incorporating the scientific, ethical, and legal bases of these activities could contribute to sticking to the laws and propose further regulations, especially those that address the cause of ongoing problems such as the overpopulation of stray dogs. A One Health/One Welfare approach in which human, animal, and environmental health are maintained in equilibrium should be at the forefront of our thinking.

Practices that are now identified as potentially harmful (e.g., tail docking, ear cropping, and animal fighting) or that can affect the health of animals in the short or long term are essential topics discussed in the Panamerican Council of Veterinary Sciences. This is the most important congress in the Americas, where more than 14 countries participate (among them Argentina, Colombia, Cuba, the United States, Peru, Brazil, Mexico, Chile, Paraguay, Venezuela, and Canada) to carry out a scientific, academic, commercial, and social exchange among institutions and professionals of veterinary medicine. In addition, this congress is organized by the Panamerican Association of Veterinary Sciences (PANVET), which brings together national representative organizations and is in charge of defining union policies, innovations, and recommendations to contribute to the protection and sustainability of the environment.

Likewise, issues related to companions and animals used for entertainment have served as a foundation to develop and reform laws and guidelines to regulate or ban activities that have been carried out for many years—due to the fact of economic or customary reasons—without deeply questioning the impact they may have on the welfare of the animals. Among the upcoming challenges is the use of noninvasive tools to assess the welfare level of the companion and entertainment animals to obtain scientific evidence to help us determine which practices should be prohibited or which can be carried out under certain conditions, as well as the development and implementation of strategies to treat animals that already were exposed to some of these practices. Research on animal welfare is needed to provide the scientific basis for decision makers on the preparation of legislation in the Latin American context.

## Figures and Tables

**Table 1:** Reasons to perform tail and ear docking, legal aspects by country, and potential implications on animal welfare.

				Legal Aspects
Procedure	Reasons to Perform	Potential Implications on AW	Country	Legislation/Regulations
**Tail docking**	Tradition [41] Prevent rabies [37] Prevent lesions during hunting [38] Increase an animal’s speed Simulate working animals and avoid paying taxes on pets [39,40] Improve hygiene in the genital area [43] Aesthetic purposes to enhance an animal’s bearing [44] Control aggressive attitudes [44]	Performed by personnel who do not have a medical license Cutting any dermal, nervous, or vascular structure entails acute pain [36] Requires anesthesia and analgesia [90] Performing this surgery in 2–5 day old puppies without anesthesia or analgesia does not avoid nociceptive signaling [38,46], and puppies can perceive pain [36] Insufficient supervision and ineffective law enforcement [9]	Argentina	National Law 14.346 on “Penalties for People who Mistreat or Commit Acts of Animal Cruelty” [91].
			Belize	Although the Animal Cruelty Act Chapter 115 mentions cruelty against companion animals; there is no available information regarding tail docking [92].
			Bolivia	Autonomous Municipal Law No. 239-316. Guidelines for the Implementation of a Comprehensive Municipal Policy to Safeguard the Welfare of Companion Animals and Public Health. It establishes sanctions against aesthetical procedures without medical purpose [93].
			Brazil	Law project 236/12. Sanctions against ear cropping and tail docking, considering them a crime [86].
			Chile	Commonly practiced surgery (represent approximately 4% of the surgeries performed on females and 9.4% on males). Performed as a practice to preserve the characteristics of pure breeds [36,45].
			Colombia	Article 6 of Law 84 from 1989 allows this kind of procedure for aesthetic reasons, and they are not considered crimes. However, Article 339A of the Colombian Penal Code states as an offense any act attempted against the life and emotional and physical integrity of animals [94].
			Costa Rica	Article 279b of Law 7451 regarding Animal Welfare imposes prison of up to one year for any person that causes intentional suffering to an animal and implicates the loss of an organ or extremity. Nonetheless, tail docking is not considered a crime, since the person is not trying to purposely cause pain [95].
			Cuba	Information not available for tail docking in dogs.
			Ecuador	Aesthetic mutilation in companion animals is banned (Article 8 of Chapter II of the Ordinance of Possession, Protection, and Control of Urban Fauna). However, it is still performed on cocker spaniel and bulldogs puppies [9].
			El Salvador	Reform of Articles 261 and 263 and the creation of The Animal Welfare Law considers aesthetic mutilations as a crime [96].
			French Guiana	Part II of the Animal Welfare Bill prohibits the amputation of a sensitive part of the body, including tail docking [97].
			Granada	Information not available.
			Guatemala	Article 61 of the decree no. 5-2017 prohibits the mutilation of ears, tails and third phalanx of companion animals, except those carried out by veterinarians in cases of justified necessity [98].
			Haiti	Information not available.
			Honduras	Article 29 of decree no. 115-2015 considers surgeries for aesthetic purposes performed without the intervention of medical personnel as a serious infraction [99].
			Mexico	The Law of Animal Protection from Mexico City [100] and states like Tamaulipas [34] and Nuevo León [101] consider caudectomy and otectomy as an act of abuse that must be punished.
			Nicaragua	Article 53 of Law no. 747: “ear and tail surgery in canines will not be considered mutilation” [102].
			Paraguay	Article 22 of Law no. 4840 prohibits mutilation or castration practices that are not for scientific, species control, or educational purposes [103].
			Panama	Article 15 of Law 70 considers it a crime to cause injury to a domestic animals [104].
			Peru	Article 27 of Law no. 30407 prohibits surgical amputations considered unnecessary or that may impede the ability to express the natural behavior of the species [105].
			Puerto Rico	Article 14 of Law no. 154: “all cosmetic surgery performed on an animal must be carried out only and exclusively by a licensed veterinarian” [106].
			Dominican Republic	Article 61 of Law no. 248-12 prohibits unnecessarily mutilating parts of a living animal [107].
			Uruguay	Article 185 of Law no. 18,471 indicates that the mutilation of an animal represents an aggravated offense [108].
			Venezuela	Article 66 of the Law for the Protection of Free and Captive Domestic Fauna establishes as a cruel act any serious organic mutilation that is not performed out of necessity and under veterinary control [109].
**Ear cropping**	In fighting dogs and shepherd breeds to reduce the incidence of bites and wounds; prevent otitis and otohematomas in dogs with droopy ears [40]	There is no scientific evidence to suggest that it prevents otitis or otohematomas [38] Negative effects such as allowing dust, dirt, and other foreign matter to reach the eardrum [53] Causes postsurgical pain because the ears are densely innervated areas that bleed easily [41]	Argentina	Kennel Club strictly forbids tail and ear clipping on all dog breeds for aesthetic reasons. It states that dogs with cropped ears and/or docked tails without a veterinary certificate are not eligible for any kind of champion title in any discipline and will not receive breeding qualification [56].
			Belize	Although the Animal Cruelty Act, Chapter 115, mentions cruelty against companion animals, there is no available information regarding ear cropping [92].
			Bolivia	Autonomous Municipal Law no. 239-316. Guidelines for the Implementation of a Comprehensive Municipal Policy to Safeguard the Welfare of Companion Animals and Public Health [93].
			Brazil	It is considered a crime and a form of animal abuse. However, performing cuts on the ears of neutered free-roaming cats is acceptable as a symbol so as not to intervene surgically on the same animal [8].
			Chile	Veterinary Medical Association (Colmevet), through its National Technical Commissions on Responsible Pet Ownership, Bioethics, and Animal Welfare, absolutely rejects cosmetic surgeries performed on animals and urges the ban of these aesthetic surgeries.
			Colombia	A practice objected to by veterinarians and animal rights activists. This situation has also led to professional usurpation when surgical procedures are performed by nonauthorized personnel [10].
			Costa Rica	Although Article 279b of Law 7451 regarding Animal Welfare imposes prison on any person that causes intentional suffering to an animal and implicates the loss of an organ or extremity, it does not mention ear cropping [95].
			Cuba	Information not available for tail docking in dogs.
			Ecuador	Article 8 of Chapter 8 of the Ordinance of Possession, Protection, and Control of Urban Fauna bans aesthetic mutilation in companion animals [9].
			El Salvador	Reform of Articles 261 and 263 and the creation of The Animal Welfare Law categorize aesthetical mutilations as crimes. However, it does not refer specifically to ear cropping or tail docking [96].
			French Guiana	Part II of the Animal Welfare Bill prohibits the amputation of a sensitive part of the body, including ear cropping and other procedures such as declawing, devocalization, and interventions aimed to modify the appearance of the animals [97].
			Granada	Information not available.
			Guatemala	Article 61 of the decree no. 5-2017 prohibits the mutilation of ears, tails and third phalanx of companion animals, except those carried out by veterinarians in cases of justified necessity [98].
			Haiti	Information not available.
			Honduras	Article 29 of decree no. 115-2015 considers surgeries for aesthetic purposes performed without the intervention of medical personnel as a serious infraction [99].
			Mexico	The Law of Animal Protection from Mexico City [100] and states such as Tamaulipas [34] and Nuevo León [101] consider caudectomy and otectomy as an act of abuse that must be punished.
			Nicaragua	Article 53 of Law no. 747: “ear and tail surgery in canines will not be considered mutilation” [102].
			Paraguay	Article 22 of Law no. 4840 prohibits mutilation or castration practices that are not for scientific, species control, or educational purposes [103].
			Panama	Article 15 of Law 70 considers it a crime to cause injury to a domestic animal [104].
			Peru	Article 27 of Law no. 30407 prohibits surgical amputations considered unnecessary or that may impede the ability to express the natural behavior of the species [105].
			Puerto Rico	Article 14 of Law no. 154: “all cosmetic surgery performed on an animal must be carried out only and exclusively by a licensed veterinarian” [106].
			Dominican Republic	Article 61 of Law no. 248-12 prohibits unnecessarily mutilating parts of a living animal [107].
			Uruguay	Article 185 of Law no. 18471 indicates that the mutilation of an animal represents an aggravated offense [108].
			Venezuela	Article 66 of the Law for the Protection of Free and Captive Domestic Fauna establishes as a cruel act any serious organic mutilation that is not performed out of necessity and under veterinary control [109].

**Table 2:** Arguments in favor and against activities that involve animals for entertainment purposes. In the cases of bull-, dog-, and cockfights; circuses; zoos; and dolphinariums points of controversy are noted as is the relevant legislation in various Latin American countries (Chile, Mexico, Argentina, and Brazil).

Activity	Arguments in Favor	Arguments Against	Country	Legislation/Regulations	Controversies
**Dog- and cockfighting**	Entertainment [199] Cultural practice [214,226]	Animals experience pain [225] Severe injuries [225] Unnecessary death [225]	Mexico	The Law of Animal Protection prohibits activities such as animal fighting.	Cockfighting is not banned [228].
			Brazil	Cockfighting and dog fighting are banned [231].	
			Colombia	Associations or institutions dedicated to dog training for entertainment, or dog fighting is prohibited [254].	
			Costa Rica	Cockfighting is banned [217].	
			Argentina	Law 14.346 establishes sanctions against activities involved in animal abuse or cruelty.	Clandestine dog racing is prohibited but dog tracks are authorized by law 12.499 [182].
			Chile	There is a proposal to prohibit cockfighting and dog fighting [233].	
			Honduras	Dogfighting is banned [232].	
			Venezuela		Cockfighting is not banned [219].
			Peru	Law 30,407 states that the training, promotion, and organization of animal fights is prohibited	Exception: bullfights, cockfights, and other acts considered as part of the culture [212].
**Bullfighting**	The cultural identity of some countries [203] Promotes tourism [13] Endorphin secretion helps relieve pain [204]	Altered behavior due to the fact of pain [206] Severe anatomical damage [207] Unnecessary sacrifice [201]	Nicaragua	Law No. 747 indicates that bullfights where the result is the death of the animal are prohibited [255].	
			Panama	Official Gazette No. 27,145-A Law 70, Article 13 bans animal races, dog fights, and bullfighting.	Horse races and cockfighting are excluded [256].
			Mexico	It is banned in six states of the country (Sonora, Guerrero, Michoacán, Veracruz, Coahuila, and Quintana Roo) [257].	
			Chile	The activity is prohibited [258].	
			Colombia		It can be performed when complying with Law 916 of 2004 [213].
			Argentina	Article 3.8 of the Law 14.346 on mistreatment and acts of cruelty to animals, of 1954. Explicitly prohibits public or private acts of animal fights, bull and heifer fights, or parodies (thereof) in which animals are killed, injured, or harassed [182].	
			Peru		Law 30,407 excludes bullfighting [212].
			Puerto Rico	Law 176 prohibits bullfights and raising bulls for fighting [259].	
			Honduras	Bullfighting shows that use of spears, swords, fire, or other objects that cause pain to the animal is prohibited [260].	According to the decree 115-2015 bullfighting and cockfighting are allowed.
**Circuses with animals**	Entertainment [235]	Confinement in narrow spaces [236] Coexistence with unknow species [242] Violent training methods [242]	El Salvador	Wildlife Conservation Law mentions that the entry, use, or mistreatment of wildlife species is prohibited in all types of shows [261].	
			Argentina	Since 2015, there have been no circuses with animal exhibitions [246].	
			Bolivia	Law 4040 indicates that the use of wild and/or domestic animals in circus shows throughout the national territory is strictly prohibited [262].	
			Colombia	Law No. 1638, includes the prohibition of the use of wild animals, whether native or exotic, of any species in fixed and traveling circus shows, regardless of their name, throughout the national territory [244].	
			Chile	A modification of existing legislation was proposed to ban it [242].	
			Peru	The forestry and wildlife law prohibits the exhibition and use of specimens of wildlife, native and exotic, in itinerant circus shows [263].	
			Nicaragua	The use of animals in circuses is banned [245].	
			Costa Rica	The entry of wild animals of any species that are part of circuses, traveling public shows, and similar organizations into the national territory is prohibited when their purpose is the public display of these animals [264].	
			Paraguay	The use of animals in circuses is banned [245].	
			Mexico	Circuses with animals are banned [240].	
