# Correction: Mota-Rojas et al. Controversial Topics in Animal Welfare in Latin America: A Focus on the Legislation Surrounding the Human-Companion Animal Relationship and Animals Used for Recreational Practices. *Animals* 2023, *13,* 1463

**DOI:** 10.3390/ani15101490

**Published:** 2025-05-21

**Authors:** Daniel Mota-Rojas, Ana Strappini, Alexandra L. Whittaker, Marcelo Ghezzi, Cristiane Gonçalves Titto, Néstor Calderón-Maldonado, Patricia Mora-Medina, Adriana Domínguez-Oliva, Jocelyn Gómez-Prado, Ismael Hernández-Ávalos, Nancy José-Pérez, Alejandro Casas-Alvarado, Agustín Orihuela

**Affiliations:** 1 Neurophysiology, Behaviour and Animal Welfare Assessment, DPAA, Xochimilco Campus, Universidad Autónoma Metropolitana, Ciudad de México 04960, Mexico; 2 Wageningen Livestock Research, Wageningen University & Research, 6708 WD Wageningen, The Netherlands; 3 School of Animal and Veterinary Sciences, Roseworthy Campus, University of Adelaide, Roseworthy, SA 5116, Australia; 4 Animal Welfare Area, Faculty of Veterinary Sciences (FCV), Universidad Nacional del Centro de la Provincia de Buenos Aires (UNCPBA), University Campus, Tandil 7000, Argentina; 5 Laboratório de Biometeorologia e Etologia, FZEA-USP, Faculdade de Zootecnia e Engenharia de Alimentos, Universidade de São Paulo, Pirassununga 13635-900, Brazil; crisgtitto@usp.br; 6 Ethology, Bioethics and Animal Welfare, Universidad La Salle, Bogotá 110231, Colombia; 7 Facultad de Estudios Superiores Cuautitlán, Universidad Nacional Autónoma de México (UNAM), Cuautitlán 54714, Mexico; 8 Facultad de Ciencias Agropecuarias, Universidad Autónoma del Estado de Morelos, Cuernavaca 62209, Mexico

## Text Correction

There was an error in the original publication [1] caused by misinterpretation of the original reference in the following paragraph: “However, nontherapeutic onychectomy is often used to avoid euthanasia or abandonment [71]”.

A correction has been made to Section 3 Companion Animals, Section 3.4 Declawing of Cats (Onychectomy), paragraph number one.

“Although nontherapeutic onychectomy was suggested to avoid euthanasia or abandonment, current research has shown that scratching behavior is an uncommon reason for shelter relinquishment and a ban on elective onychectomy did not increase shelter relinquishment or euthanasia [71]”.

The authors state that the scientific conclusions are unaffected. This correction was approved by the Academic Editor. The original publication has also been updated.
